# A Pressure-Insensitive Self-Attachable Flexible Strain Sensor with Bioinspired Adhesive and Active CNT Layers

**DOI:** 10.3390/s20236965

**Published:** 2020-12-05

**Authors:** Minho Seong, Insol Hwang, Joosung Lee, Hoon Eui Jeong

**Affiliations:** Department of Mechanical Engineering, Ulsan National Institute of Science and Technology (UNIST), Ulsan 44919, Korea; sung710uio@unist.ac.kr (M.S.); ihwang@unist.ac.kr (I.H.); sunmier@unist.ac.kr (J.L.)

**Keywords:** adhesives, bioinspired microstructures, carbon nanotubes, flexible sensors, strain sensors

## Abstract

Flexible tactile sensors are required to maintain conformal contact with target objects and to differentiate different tactile stimuli such as strain and pressure to achieve high sensing performance. However, many existing tactile sensors do not have the ability to distinguish strain from pressure. Moreover, because they lack intrinsic adhesion capability, they require additional adhesive tapes for surface attachment. Herein, we present a self-attachable, pressure-insensitive strain sensor that can firmly adhere to target objects and selectively perceive tensile strain with high sensitivity. The proposed strain sensor is mainly composed of a bioinspired micropillar adhesive layer and a selectively coated active carbon nanotube (CNT) layer. We show that the bioinspired adhesive layer enables strong self-attachment of the sensor to diverse planar and nonplanar surfaces with a maximum adhesion strength of 257 kPa, while the thin film configuration of the patterned CNT layer enables high strain sensitivity (gauge factor (GF) of 2.26) and pressure insensitivity.

## 1. Introduction

Recently, flexible tactile sensors that can transform mechanical stimuli into electrical or optical signals have been actively developed as a key component of emerging human–robot interactive systems [1,2], wearable electronics [3,4,5], healthcare devices [6,7], and prosthetics [8,9]. For the successful application of flexible mechanical sensors in these innovative systems, they should have high sensitivity over a specific detection range on diverse planar and even nonplanar target objects [3,7,10]. To achieve this requirement, low-dimensional nanomaterials such as carbon nanotubes (CNTs) [11,12], nanowires [13,14,15,16], nanoparticles [17,18,19], and graphene [20,21,22] have been utilized as active sensing components of flexible sensors based on different transduction modes of capacitance [22], piezoelectricity [16], piezoresistivity [23], and triboelectricity [12,24], owing to their excellent mechanical, electrical, and optical properties. Interestingly, when nanomaterials are incorporated into specific microstructures such as micropillars [25,26], microdomes [27], micropyramids [28,29], and microwrinkles [30], the sensing performance of flexible tactile sensors is significantly altered when compared with that of nanomaterial-based simple thin-film sensors. This is because microstructures with specific topographies induce stress concentrations and exhibit unique force-displacement behaviors under the influence of specific mechanical stimuli [27,31].

To enhance the sensing performance of flexible tactile sensors, close conformal contact with the target substrates is essential [32,33,34]. Even if the sensors have outstanding intrinsic sensing capabilities, in the absence of close conformal contact with target objects, the sensors cannot properly detect the mechanical deformations of the objects, thereby drastically reducing the device sensitivity [11]. Furthermore, unstable contact formation at the sensor–target interface degrades the reliability and repeatability of the sensor [35]. However, active nanomaterials coated over flexible sensors hinder the conformal contact of the device with the substrate owing to the surface roughness of the coated nanomaterials [36]. Layers with designed microstructures incorporated into the sensor for sensitivity enhancement also disturb the formation of intimate physical contact between the device and the target substrate [37]. Thus, flexible sensors are typically fixed over the substrates using additional adhesive tapes [38], adhesive chemicals [39], and mechanical clampers [40]. The contacts and interfaces formed by conventional chemical adhesives and mechanical clampers are typically untidy, complicated, contaminated and bulky. Ultrathin planar sensors can conformably attach to various target substrates, including skin, without using additional adhesives by reducing their thickness to harness van der Waals interactions [41]. However, they are mechanically less durable and have limited adhesion strengths [42]. 

Facile differentiation of the different mechanical stimuli of tensile strain and normal pressure is also a critical requirement for the practical application of flexible tactile sensors [33]. Although previous flexible tactile sensors have demonstrated high sensitivity to strain and pressure, electrical output signals responding to these input signals are similar and indistinguishable from each other [43]. Accordingly, the decoupling of strain and pressure is highly challenging with most of the previously reported flexible tactile sensors. Recent studies demonstrated that strain-insensitive pressure sensors can be developed by utilizing specific microscale topographies that maximize pressure sensitivity and minimize strain responsiveness (e.g., micropyramid) [44]. On the other hand, pressure-insensitive flexible strain sensors have rarely been reported. Recently, Oh et al. suggested a novel flexible strain sensor that can selectively detect strains [45]. However, its self-adhesion behavior with quantitative evaluation was not reported. In addition, it showed nonlinear piezoresistivity for applied strains. Overall, despite recent advances, self-attachable flexible strain sensors with outstanding sensing performance and strong adhesion strengths, as well as the capability to decouple pressure and strain, are rarely explored (Table S1). For example, previous studies have reported strain sensors that can exhibit pressure (or strain) insensitivity. However, they exhibited limited adhesion capability against target substrates [45,46]. On the other hand, strain sensors with enhanced adhesion strengths showed a limited gauge factor (GF) or strain range [47,48]. Additionally, they could not decouple the strain from normal pressure. 

Herein, we present a self-attachable, high-performance, pressure-insensitive strain sensor that can firmly adhere to target substrates and transduce tensile strain with high sensitivity. The proposed sensor is mainly composed of a bioinspired micropillar adhesive layer and a selectively coated active multiwalled CNT (MWCNT) layer. The uniformly coated thin film configuration of the active MWCNT layer enables a highly sensitive transformation of an external strain into electrical signals based on piezoresistive transduction while minimizing responsiveness to normal pressure. The micropillar layer enables an intimate and strong mechanical coupling with target surfaces (pull-off strength of 257 kPa) without using additional chemical adhesives and mechanical clips, which contributes to enhancing the sensing performance. We demonstrate that the proposed sensor exhibits excellent differentiation of applied strain and pressure with high strain sensitivity (GF of 2.26), fast response (90 ms), and high durability (>1000 cycles) while maintaining intimate and robust contact with diverse planar and nonplanar substrates.

## 2. Materials and Methods

### 2.1. Fabrication of the Pressure-Insensitive Self-Attachable Flexible Strain Sensor

The Si master mold with the negative pattern of micropillars with protruding tips was prepared through photolithography (Figure S1) [49]. First, a dehydrated Si wafer was spin-coated with the lift-off resist (LOR 30B, Microchem Corp., USA), followed by baking at 200 °C for 30 min. Subsequently, a photoresist (SU-8 3010, Microchem Corp., USA) was spin-coated onto the LOR layer and baked at 95 °C for 3 min. The bilayer of LOR/SU-8 was then exposed to UV (λ = 365 nm, dose = 200 mJ cm^−2^) using a photomask with microdot patterns. After UV exposure, additional baking (95 °C for 2 min) was carried out. Then, the SU-8 layer was developed using an SU-8 developer (Microchem Corp., USA) for 5 min to yield a negative micropillar array. The LOR layer under the hole pattern was selectively and gradually removed with an LOR developer (AZ 300 MIF, Merck, Germany) for 2 min to form an undercut (length: 4 µm) for a negative tip shape. The fabricated Si master was passivated with C_4_F_8_ gas for surface hydrophobization. A polydimethylsiloxane (PDMS) mixture containing 9.1 wt% of curing agent (Sylgard 184B, Dow Corning, USA) in prepolymer (Sylgard 184A, Dow Corning, USA) was dispensed over the master. The PDMS mixture was thermally cured in a convection oven at 70 °C for 2 h. After curing, the PDMS replica with micropillar arrays having protruding tips was demolded from the master. For the preparation of the MWCNT solution, COOH-functionalized MWCNTs (RND Korea, Republic of Korea) with an outer diameter of 20–30 nm and a length of 10–30 µm were dispersed in ethanol (0.3 wt %), followed by sonication for 1 h. To enhance the adhesion of the MWCNTs with PDMS, (3-aminopropyl)triethoxysilane (APTES) was applied to the bottom surface of the PDMS micropillar array, while the tip surface of the micropillars was covered with a glass [50]. Subsequently, the MWCNT solution was spray-coated onto the entire surface of the PDMS replica, including the micropillars with protruding tips. The PDMS replica coated with the MWCNT solution was dried at 70 °C for 1 h to remove the remaining solvent. Finally, the MWCNTs coated over the tips of the micropillars were selectively removed using an adhesive tape, yielding a pressure-insensitive self-attachable flexible strain sensor.

### 2.2. Surface Analysis

High-resolution SEM images of the microstructures and MWCNT percolation networks were obtained using an S-4800 microscope (Hitachi, Japan). Before imaging, a thin Pt layer (thickness of 5 nm) was deposited on the samples by metal sputtering (K575X sputter coater, Quorum Emitech, UK) to prevent charging effects.

### 2.3. Evaluation of the Adhesion Behavior of the Self-Attachable Flexible Strain Sensor

The adhesion strengths were evaluated using custom-built equipment, with a fixed stage and a motorized part above the stage. The motorized part, directly connected to a load cell (KTOYO, Republic of Korea), was movable along the vertical direction. The square samples (area: 1 × 1 cm^2^) were mounted on the horizontal surface of the motorized part with the microstructures of the samples facing down. For the measurements, the mounted samples were brought in contact with the target substrates on a fixed stage with a controlled preload. Then, an out-of-plane displacement was applied along the vertical direction (speed: 1 mm s^−1^), until the samples were detached from the substrates. For each sample, the measurements were repeated 10 times, and the average values were presented.

### 2.4. Characterization of the Piezoresistive Sensing Behavior of the Pressure-Insensitive Flexible Strain Sensor

The sheet resistance of the deposited MWCNT percolation networks was measured using a four-point probe method with a surface resistivity meter (CMT-SR1000N, Advanced Instrument Technology, Korea). The electrical resistance changes were measured using a two-probe method with a source meter (model 6430, Keithley, USA) while applying mechanical stimuli. Two opposite sides of the rectangular samples (initial length of 2 cm and thickness of 1 mm) were fixed by mechanical clamping and connected with electrodes (copper wire) using a silver paste to reduce the contact resistance. The tensile and normal stresses were applied separately or simultaneously using custom-built equipment. The equipment consisted of two motorized parts of a horizontally movable clamper and a vertically movable load cell (KTOYO, Korea). The applied voltage for the resistance measurement was 20 V.

## 3. Design and Fabrication of the Pressure-Insensitive Self-Attachable Flexible Strain Sensor

**Figure 1:**
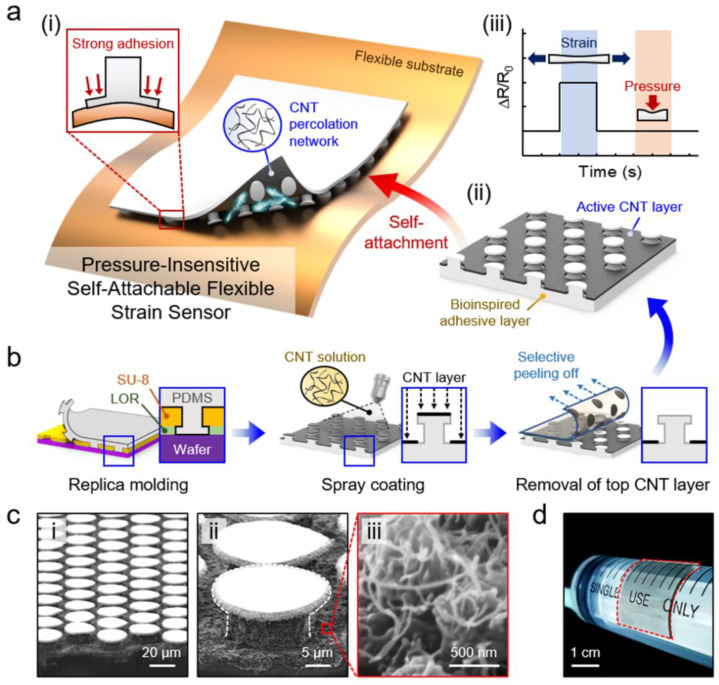
Design of the pressure-insensitive self-attachable flexible strain sensor. (**a**) (**i**,**ii**) Schematic illustration showing the pressure-insensitive strain sensor with an active multiwalled carbon nanotube (MWCNT) layer and a bioinspired adhesive micropillar layer. (**iii**) Strain-sensitive and pressure-insensitive properties of the sensor. (**b**) Fabrication procedure of the strain sensor. (**c**) (**i**) SEM images of the fabricated strain sensor with (**ii**) micropillar layer and (**iii**) MWCNT layer. (**d**) Photograph of the self-attachable strain sensor firmly attached to the curved surface of a syringe.

Figure 1a shows a conceptual schematic of the pressure-insensitive self-attachable strain sensor proposed in this study. The sensor has two main device components: a selectively coated percolating MWCNT layer and a mushroom-shaped micropillar array (Figure 1a-i). The MWCNTs are deposited on the bottom surface of the strain sensor, except for the micropillars (Figure 1a-ii). When a tensile strain is applied to the MWCNT layer deposited on the sensor, microscale cracks occur within the MWCNT percolation network, and the distance between the networks increases with an increase in strain, resulting in large changes in the electrical resistance [45]. On the other hand, the application of normal pressure does not significantly alter the MWCNT percolation network because the MWCNT layer is very thin (thickness of ~200 nm), and thus the deformation of the layer under pressure is highly limited. In addition, the micropillars with protruding tips shield the active MWCNT layer from the applied pressure. Therefore, the proposed sensor exhibits high sensitivity to strain while showing negligible responses to pressure (Figure 1a-iii). 

Although the deposited MWCNT layer acts as an active component of the sensor, it hinders the conformal adhesion of the sensor to the target substrate [51]. In this case, the sensing performance and measurement reliability can significantly deteriorate. To address this issue, we integrated bioinspired adhesive structures into the strain sensor (Figure 1a). Some living creatures such as gecko lizards and beetles have dense microscopic hairy structures with protruding tips on their feet [52,53,54]. These intriguing hairy structures impart their feet with strong dry adhesion capability by maximizing the van der Waals interactions [55,56]. In particular, the protruding tips play a critical role in maximizing the adhesion strength by enhancing the real contact area and uniformly distributing the contact stresses [57,58,59]. We harnessed the nature-inspired micropillar structure comprising protruding ends in our sensor design to equip the sensor with strong self-attachable capability.

Figure 1b shows the fabrication procedure of the self-attachable strain sensor. First, a PDMS pad with micropillars was generated by a replica molding technique (see Figure S1 for details). Then, an MWCNT solution (0.3 wt % in ethanol) was spray-coated over the PDMS surface with micropillars. The MWCNTs deposited over the tips of micropillars were selectively removed by using an adhesive tape, as the MWCNTs on the tips would impede the adhesion of the micropillar array. Figure 1c shows the generated adhesive micropillar array with a stem diameter of 15 µm, a tip diameter of 23 µm, a height of 10 µm, and a pitch of 30 µm. MWCNTs were selectively deposited on the bottom surface of the sensor, except for the micropillars, forming percolation networks. As shown in Figure 1d, intimate adhesion of the fabricated flexible strain sensor to the curved surface of a syringe occurred without using additional adhesive tapes owing to the intrinsic adhesive nature of the micropillar array.

## 4. Adhesion Behavior of the Self-Attachable Flexible Strain Sensor

**Figure 2:**
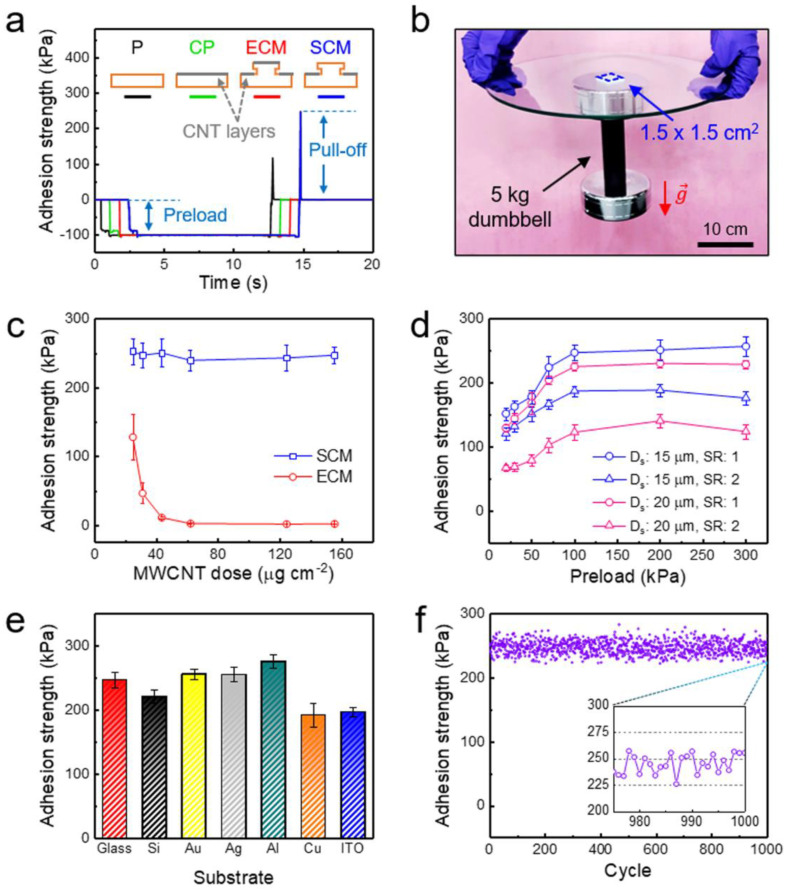
Adhesion behavior of the strain sensor. (**a**) Measured adhesion strengths of the planar polydimethylsiloxane (PDMS; P), MWCNT-coated planar PDMS (CP), entirely MWCNT-coated PDMS micropillars (ECM), and selectively MWCNT-coated PDMS micropillars (SCM) (preload: 100 kPa, pulling rate: 1 mm s^−1^). (**b**) Photograph showing a 5 kg dumbbell attached to a glass plate via the self-attachable strain sensor (area: 1.5 × 1.5 cm^2^). (**c**) Adhesion strengths of the ECM and SCM sensors as a function of coating dose of the MWCNTs (preload: 100 kPa, pulling rate: 1 mm s^−1^). (**d**) Adhesion strengths of the SCM sensors with different pillar stem diameters (*D_s_*: 15 and 20 µm; spacing ratio (SR): 1 and 2). (**e**) Adhesion strengths of the SCM sensors with a pillar *D_s_* of 15 µm and an SR of 1 against different substrates (preload: 100 kPa, pulling rate: 1 mm s^−1^). Error bars in (**c**–**e**) represent the standard deviations, and each test was repeated 10 times. (**f**) Adhesion durability of the SCM sensor after repeated cycles of attachment and detachment.

The self-attachable capability of the flexible strain sensor was evaluated by measuring the pull-off adhesion strengths of the sensor on a flat glass substrate. Figure 2a shows the measured adhesion strengths of the four different devices: planar PDMS (P), MWCNT-coated planar PDMS (CP), PDMS micropillars coated with MWCNTs over the entire surface (ECM), and PDMS micropillars selectively coated with MWCNTs on the bottom surface (SCM). The planar PDMS device without an MWCNT layer (P) showed a fair adhesion strength of 100 kPa due to the soft elastomeric nature of the PDMS. However, when an active MWCNT layer was coated over the planar PDMS device (CP), its adhesion strength reduced to almost zero, indicating that the device cannot adhere to the target substrate without using additional adhesive tapes. When the entire surface of the PDMS micropillars was coated with the CNT layer (ECM), the micropillars also exhibited negligible adhesion strengths. On the other hand, the PDMS micropillars selectively coated with the CNT layer on the bottom surface, in which the tip surface was not coated with the CNT layer (SCM), showed a significantly enhanced adhesion strength of ~250 kPa. Therefore, the SCM-based sensor possessed remarkable self-attachability to target substrates without the use of additional adhesives or tapes. Indeed, the proposed SCM sensor was demonstrated to support a heavy dumbbell of 5 kg in weight from a glass substrate (Figure 2b). We further investigated the adhesion strengths of the micropillars with and without the MWCNT layer on the tip as a function of the coating dose of the CNT layer (Figure 2c). With an increase in the CNT coating dose, the adhesion strength of the ECM rapidly decreased and reached almost “zero”. By contrast, SCM maintained its strong adhesion strength while the sheet resistance significantly decreased from ~10^7^ to ~10^4^ Ω sq^−1^, when the coating dose of the MWCNT layer was increased (Figure S2a). Based on the measured sheet resistance and thickness of the MWCNT layer as a function of the coating dose of the MWCNT, we evaluated the conductivity (calculated by 1/sheet resistance × 1/thickness) of the MWCNT layer. As shown in Figure S2b, the MWNCT layer exhibited a saturated conductivity of 440.4 S m^−1^ at a coating dose of 155.3 μg cm^−2^. According to a previous study, the conductivity of the CNT layers formed by the spray coating becomes nearly independent of thickness, if the percolation network is sufficiently formed [60].

We also investigated the effect of the micropillar geometries on the adhesion strengths. Figure 2d shows the adhesion strengths of the selectively coated CNT micropillars (SCM) with four different geometries: micropillars with a stem diameter (*D_s_*) of 15 μm and a spacing ratio (ratio of spacing to stem diameter, SR) of 1, micropillars with a *D_s_* of 15 μm and an SR of 2, micropillars with a *D_s_* of 20 μm and an SR of 1, and micropillars with a *D_s_* of 20 μm and an SR of 2. A higher pillar density with a lower *D_s_* and a lower SR can lead to a higher adhesion strength. However, *D_s_* of less than 15 μm can deteriorate the structural stability of the pillars, while it requires much higher fabrication costs. In addition, an SR below 1 can lead to lateral collapse between adjacent pillars. As expected, the micropillar array with a *D_s_* of 15 μm and an SR of 1 exhibited the highest adhesion strength, because it had the highest pillar density among the different samples. All the samples showed increased adhesion strengths with an increase in the preload and exhibited adhesion saturation at a preload of ~100 kPa (Figure 2d). In addition to the glass substrate, the SCM sensor exhibited strong self-attachability to a wide range of substrates including Si, Au, Ag, Al, Cu, and ITO (Figure 2e). The SCM sensor also showed high adhesion strengths with glass substrates having different surface roughness (root mean square: 0.05, 0.33, 1.89, and 5.18 µm) (Figure S3). Furthermore, the strong self-attachable capability was maintained over 1000 cycles of attachment and detachment testing without exhibiting signs of adhesion degradation (Figure 2f). These results demonstrated that the flexible strain sensor with a selectively coated active CNT layer can firmly adhere to diverse target substrates and intimately interface with them, enabling the precise detection of the mechanical deformations of the substrates.

## 5. Sensing Behavior of the Self-Attachable Flexible Strain Sensor

**Figure 3:**
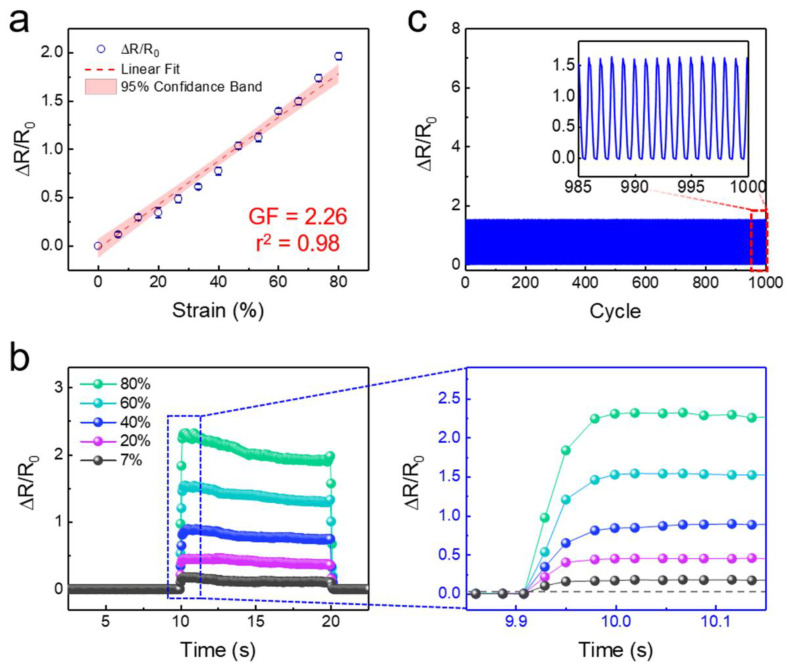
Strain sensing behavior of the self-attachable strain sensor. (**a**) Relative resistance change as a function of applied strain. The average values and error bars are based on five measurements. Error bars represent the standard deviation (<0.05). The standard error of the gauge factor (GF) is 0.024. (**b**) Time-resolved measurement of the relative resistance at different applied strains. (**c**) Durability of the sensor after repeated cycles of an applied strain (60%) (frequency: 0.067 Hz, duration of each cycle: 15 s).

A GF, which is defined as GF = (Δ*R/R*_0_)/(Δ*L/L*_0_), represents the performance of the strain sensors. Here, *R* is the electrical resistance and *L* is the length of the strain sensor. Figure 3a shows the relative resistance change of the self-attachable strain sensor (*D_s_*: 15 μm and SR: 1) as a function of the applied strain from 0 to 80%. The maximum strain range was set to 80%, since PDMS has an elongation at break between 80% and 100% of tensile strain [61]. As shown, the sensor exhibited a highly linear change in the relative resistance under a wide in-plane tensile strain range of 0–80%, with a GF of 2.26. According to previous studies, active nanomaterials with lower conductivity can lead to a higher GF. Thus, the GF of our strain sensor could be further enhanced by optimizing the conductivity of the MWCNT layer (Figure S2) [62]. The pillar density can also affect the GF of the sensor, as it affects the area and conductivity of the MWCNT layer [63]. According to our measurements, no apparent difference in GF was observed among the SCM sensors with four different pillar geometries (Figure S4). It seems that a small difference of *D_s_* (15 and 20 μm) resulted in a negligible difference in GF. Further studies are required to optimize the geometry of pillars. The application of in-plane compressive strain on the MWCNT percolation network induced a reduction in the electrical resistance because of the increased contacts between MWCNTs under in-plane compressive strain (Figure S5). It is noted that the application of in-plane compressive strain over 0.1 resulted in the in-plane buckling of PDMS. Figure 3b shows the time-lapse electrical responses of the strain sensor for different strains from 7% to 80%. As shown, the strain sensor exhibited immediate responses (<90 ms) and relaxation (<150 ms) for all the applied strain ranges. When a relatively high tensile strain (>60 %) was applied to the sensor, a slight overshoot followed by the temporal decay of the relative resistance was observed. This is caused by the stress relaxation and viscoelastic behavior of PDMS under tensile strain [64]. When a tensile strain is applied to the sensor, the stress is transferred to the PDMS and MWCNT layers, resulting in a rearrangement of the MWCNTs. Meanwhile, the internal structure of PDMS releases sudden stress by immediate mechanical deformation. This induces a gentle restoration of the conductive paths between MWCNTs, resulting in the temporal decay of resistance. The strain sensing behavior of the proposed strain sensor was highly robust and durable (Figure 3c). With repeated cycles of the strain loading and unloading durability tests using 60% of an applied strain, the sensor showed a stable and uniform change in the relative resistance over 1000 cycles. These results showed that the sensor not only strongly interfaces with the target substrate but also detects mechanical strains with reliable sensitivity and durability. Note that although it is rare for PDMS to be permanently deformed below an 80 % strain due to its viscoelastic nature, the MWCNT percolation networks can be permanently deformed under the application of fixed strains over a long period of time [65]. Temperature and humidity can also affect the performance of the sensor [65,66]. Further studies are required to study the durability of the sensor under long-term fixed strain or varying environmental conditions.

**Figure 4:**
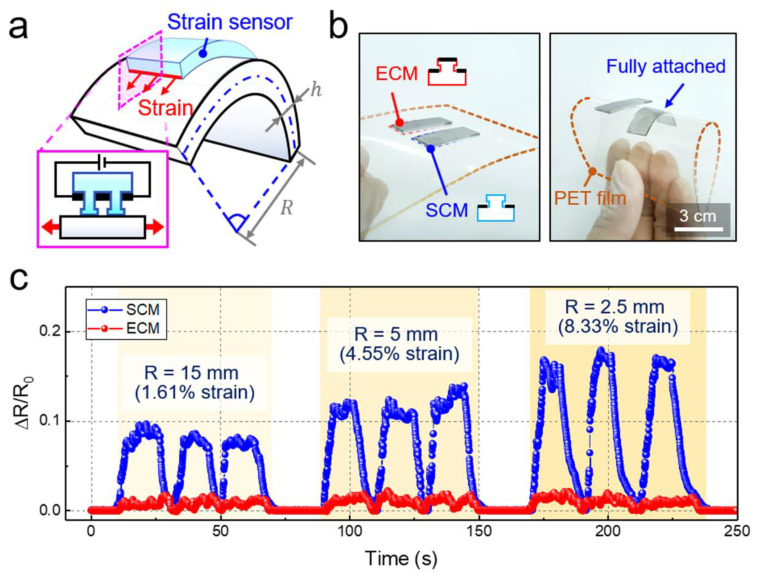
Comparison of the sensing performances of the self-attachable and non-attachable strain sensors. (**a**) Schematic illustration showing the working principle of the self-attachable strain sensor under bending stress. (**b**) Photographs showing the adhesion and bending behavior of the entirely MWCNT-coated micropillar (ECM) and selectively MWCNT-coated micropillar (SCM) strain sensors attached on a PET film under bending. (**c**) Time-resolved changes in the relative resistance measured by the ECM and SCM sensors for different bending radii (15, 5, and 2.5 mm).

Because of the flexible nature of the CNTs and the PDMS used for the strain sensor as well as the self-attachable capability of the sensor, the proposed sensor also perceived bending stresses (Figure 4a). Figure 4b shows the entirely CNT-coated (ECM) and selectively CNT-coated (SCM) sensors that were placed on a thin PET film. Without bending, both the ECM and SCM sensors maintained adhesion on the PET film. However, when the PET film was highly bent, the ECM sensor could not maintain its attachment to the film due to its negligible adhesion strength. By contrast, the SCM sensors firmly adhered to the substrate and conformed to the bend of the PET substrate owing to its strong self-attachability. Although the uncoated planar backside of the SCM sensors could be attached to the PET surface, they were also easily peeled off under bending (Figure S6). Figure 4c shows the electrical behavior of the two different sensors under different bending radii (R) of 15, 5, and 2.5 mm. As expected, the ECM sensors could not properly detect the bending of the PET film due to delamination from the substrate. By contrast, the self-attachable SCM strain sensor could sensitively perceive different bending stresses applied to the PET substrate (Figure 4c). We performed additional experiments that can demonstrate the monitoring application of human physical activities with the SCM sensor (Figure S7). Based on the bioinspired adhesive microstructures, the SCM strain sensor could be firmly attached to the skin of the wrist. When the wrist was bent, the relative electrical resistance was rapidly increased, which indicates that the tensile strain caused by the wrist bending was immediately transmitted to the sensor. When the wrist was back to the original unbent state, the resistance was returned to the initial value. The SCM sensor exhibited stable and reproducible electrical behavior during the repeated bending of the wrist.

**Figure 5:**
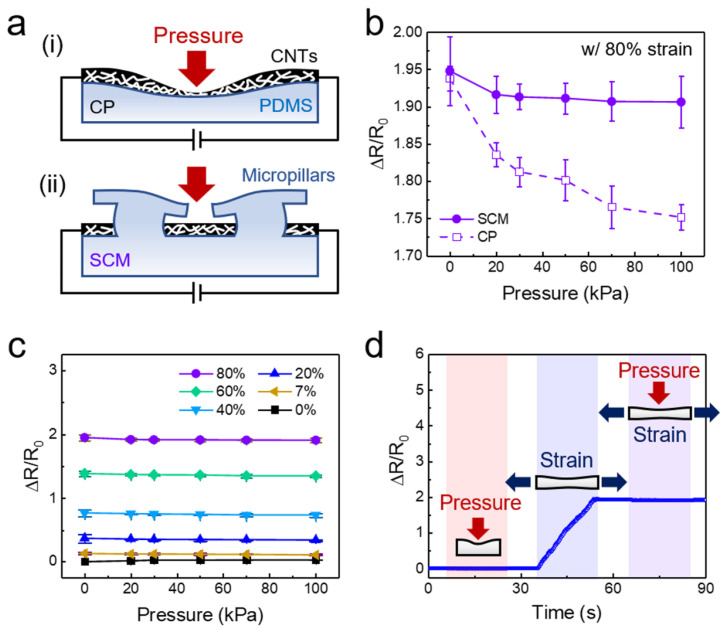
Strain-sensitive and pressure-insensitive property of the strain sensor. (**a**) (**i**) Schematic illustration showing the working principle of the CP sensor. (**ii**) Schematic illustration showing the pressure-insensitive working principle of the selectively MWCNT-coated micropillar (SCM) strain sensors. (**b**) Relative resistance changes measured by the CP and SCM sensors as a function of pressure (applied strain = 80%). (**c**) Relative resistance changes measured by the SCM sensor as a function of pressure for different strains (0–80%). The average values and error bars in (**b**,**c**) are based on five measurements. Error bars represent the standard deviation. (**d**) Time-resolved measurement of an applied pressure (100 kPa) and a strain (80%) with the SCM sensor.

Many previous flexible tactile sensors produce similar electrical responses under normal pressure and tensile strain, which significantly limits their practical application [43,67]. The SCM-based sensor proposed in this study can address this issue by harnessing the selectively coated MWCNT layer and the micropillar layer. The minimally deformable thin configuration (thickness: ~200 nm) of the coated MWCNT layer minimizes the changes in the percolation networks and the electrical resistance. However, pressure applied over the MWCNTs on elastomeric PDMS results in small mechanical deformation of the MWCNT layer, thereby inducing changes in the electrical resistance (Figure 5a-i). The micropillars with protruding tips also serve as physical shields for the MWCNT layer against the applied pressure, and thus, the pressure responsiveness of the sensor is minimized (Figure 5a-ii). Indeed, the MWCNT-coated planar (CP) sensor showed relatively larger pressure responsiveness and clear changes in the resistance with increasing pressure, while the SCM sensor showed minimal pressure responsiveness (Figure 5b). Figure 5c shows the electrical resistance change of the SCM sensor under different strains and pressures. As shown, although the SCM sensor sensitively responded to the applied strain from 0 to 80%, it did not exhibit any noticeable responsiveness to the normal pressure ranging from 0 to 100 kPa. The time-lapse measurements of the relative resistance further demonstrated the pressure-insensitive and strain-sensitive property of the SCM sensor (Figure 5d). An initial application of 100 kPa in pressure to the sensor did not induce any noticeable changes in the resistance. However, when an 80% strain was applied to the sensor, a linear increase in the resistance was observed, demonstrating the decoupling capability of strain and pressure. Subsequent application of 100 kPa pressure while maintaining the 80% strain did not result in any further change in the electrical resistance. These results clearly demonstrated that the proposed SCM sensor has an intriguing pressure-insensitive and strain-sensitive property, which enables the facile differentiation of tensile strain and normal pressure. To evaluate the reproducibility of sensing performance, we prepared five SCM sensors (coating dose of MWCNTs: 155.3 μg cm^−2^) and compared their GF, adhesion, and pressure insensitivity properties (Figure S8). The measured adhesion strengths (245.1–251.1 kPa), GF for tensile strains up to 80% (2.16–2.28), and relative resistance changes for a normal pressure of 100 kPa (−0.028 to −0.024) were all highly reproducible among the different SCM sensors.

## 6. Conclusions

In summary, we proposed a new type of strain sensor that can strongly and conformably adhere to target substrates and selectively detect applied strains with high sensitivity. The intriguing sensing performance was enabled by integrating a selectively deposited MWCNT layer and a bioinspired adhesive micropillar array into the sensor device. The thin MWCNT layer selectively deposited on the bottom surface of PDMS enabled the selective detection of applied tensile strains while minimizing the responsiveness to normal pressures. The micropillar array with protruding tips equipped the sensor with strong self-attachability. Simultaneously, the micropillars prevented the normal pressures from reaching the active MWCNT layer, and thus, the sensor was insensitive to pressure stimuli. The self-attachability and strain–pressure decoupling ability of the proposed sensor is not easily achievable with other flexible mechanical sensors. The GF of 2.26 and the maximum strain range of 80% are acceptable for a wide range of applications of flexible mechanical sensors, including electronic skins [68], healthcare devices [69], and structural monitoring systems [70], where robust adhesions between the flexible sensors and various target substrates (such as glass, metal, semiconductor, and skin) are prerequisite. We believe that our flexible strain sensor with strong self-attachability, sensitive strain responsiveness, and pressure insensitivity will contribute to the development of more advanced flexible mechanical sensors and electronic skins.

## Supplementary Materials

The following are available online at https://www.mdpi.com/1424-8220/20/23/6965/s1, Table S1: Comparisons of the GFs, maximum tensile strains, pressure insensitivities (relative resistance changes under normal pressures), and adhesion strengths between the developed sensor in this work and those in similar previous studies, Figure S1: Schematic of the fabrication procedure of the pressure-insensitive self-attachable flexible strain sensor, Figure S2: Sheet resistance and MWCNT layer thickness (**a**) and conductivity (**b**) of the self-attachable flexible strain sensors as a function of the coating dose of the MWCNTs, Figure S3: Adhesion strengths of SCMs (coating dose: 155.3 μg cm^−2^) against glass substrates with different roughness (root mean square (RMS): 0.05, 0.33, 1.89, and 5.18 µm), Figure S4: GFs of SCMs with different pillar stem diameters and SRs, Figure S5: Relative resistance change as a function of applied in-plane tensile and compressive strain, Figure S6: Attachment behavior of the SCM strain sensors. (**a**) Schematic illustration showing the different attachment modes of the SCM sensors to a PET substrate using the planar backside (**i**) and the frontside (**ii**) of the SCMs. (**b**) Photograph showing the different adhesion behaviors of the SCM sensors attached to a flexible PET substrate using its backside and frontside under bending stress, Figure S7: Demonstration of a self-attachable strain sensor for the monitoring application of human physical activities, Figure S8: Adhesion strengths, relative resistance changes for a normal pressure (100 kPa), and GFs for tensile strains (range: 0 to 80%) of five SCM samples (coating dose of MWCNTs: 155.3 μg cm^−2^).
