# Supplementary material for: A Pressure-Insensitive Self-Attachable Flexible Strain Sensor with Bioinspired Adhesive and Active CNT Layers
Source: Sensors (Basel). 2020 Dec 5;20(23):6965. doi: 10.3390/s20236965 (PMC7730429; doi:10.3390/s20236965)
Supplement: Supplementary file 1 [file sensors-20-06965-s001.pdf]

Supporting Information

A Pressure-Insensitive Self-Attachable Flexible Strain Sensor with Bioinspired Adhesive and Active CNT Layers

Minho Seong †, Insol Hwang †, Joosung Lee and Hoon Eui Jeong *

Department of Mechanical Engineering, Ulsan National Institute of Science and Technology (UNIST), Ulsan 44919, Korea; sung710uio@unist.ac.kr (M.S.); ihwang@unist.ac.kr (I.H.); sunmier@unist.ac.kr (J.L.)

* Correspondence: hoonejeong@unist.ac.kr (H.E.J.)

† These authors contributed equally to this work.

Table S1. Comparisons of the GF, the maximum tensile strains, pressure insensitivities (relative resistance changes under normal pressure), and adhesion strengths between the developed sensor in this work and those in similar previous studies.

Material	GF	Maximum tensile strain (%)	Pressure insensitivity (Applied pressure)	Adhesion strength (kPa) (Target substrate)	References
MWCNT[a]/ PDMS[b]	56	70	–0.010 (100 kPa)	N/A	[1]
AgNW[c]/PDMS /Silica aerogel	1.57	100	–0.014 (79 kPa)	N/A	[2]
AgNP[d]/PDMS/ VS[e]	767	1	N/A	18 (skin)	[3]
AgNP/Graphene/ PVA[f]/PDA[g]	0.93	315	N/A	7.6 (glass)	[4]
MWCNT/PDMS	2.26	80	–0.026 (100 kPa)	257 (glass)	This work

[a]Abbreviation: MWCNT, multi-walled carbon nanotube
[b]Abbreviation: PDMS, polydimethylsiloxane
[c]Abbreviation: AgNW, silver nanowire
[d]Abbreviation: AgNP, silver nanoparticle
[e]Abbreviation: VS, vinylsiloxane
[f]Abbreviation: PVA, polyvinyl alcohol
[g]Abbreviation: PDA, polydopamine

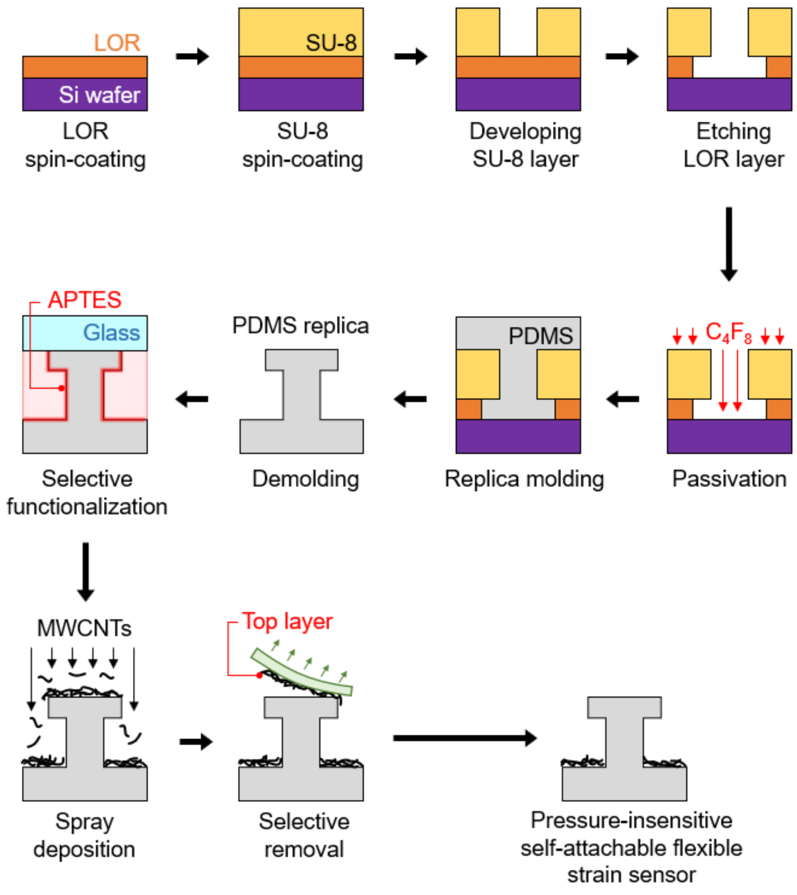

Figure S1. Schematic of the fabrication procedure of the pressure-insensitive self-attachable flexible strain sensor.

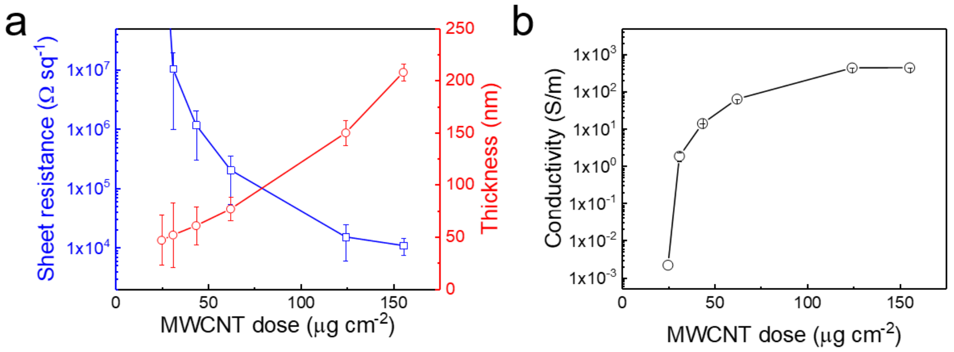

Figure S2. Sheet resistance and MWCNT layer thickness (**a**) and conductivity (**b**) of the self-attachable flexible strain sensors as a function of the coating dose of the MWCNTs. The average values and error bars are based on five measurements. The error bars represent the standard deviations.

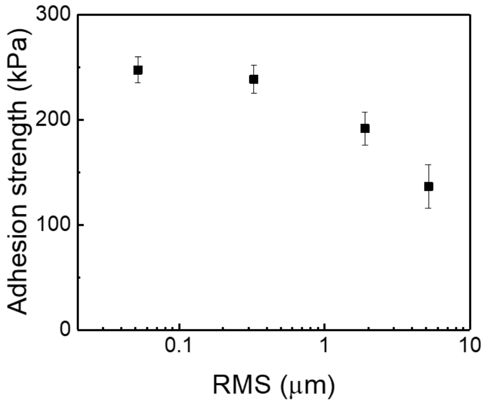

Figure S3. Adhesion strengths of SCMs (coating dose: 155.3 μg cm^{-2}) against glass substrates with different roughness (root mean square (RMS): 0.05, 0.33, 1.89, and 5.18 μm). Each glass substrate was prepared by roughening the surface using sandpaper. The average values and error bars are based on 10 measurements. The error bars represent the standard deviations.

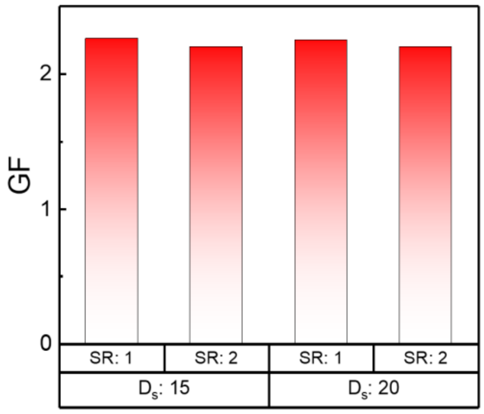

Figure S4. GFs of SCMs with different pillar stem diameters and SRs. The D_s are 15 and 20 μm, and The SRs are 1 and 2.

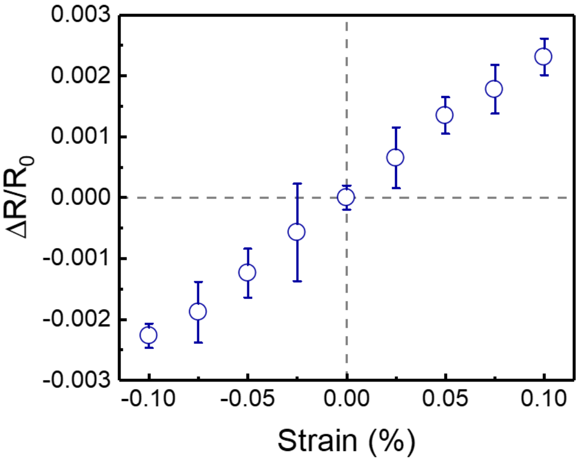

Figure S5. Relative resistance change as a function of applied in-plane tensile and compressive strain. The average values and the error bars are based on five measurements. The error bars represent the standard deviations.

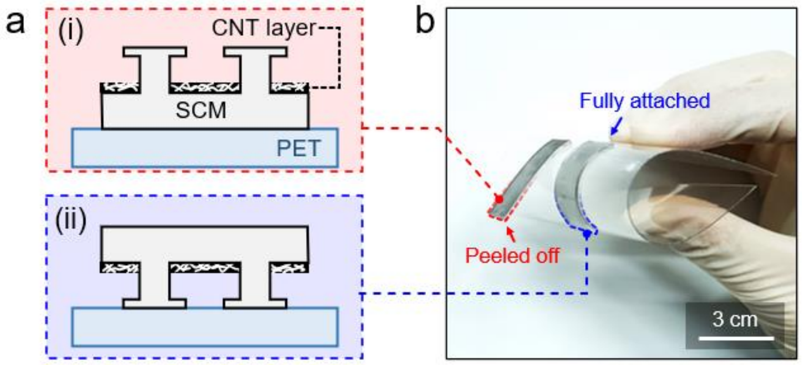

Figure S6. Attachment behavior of the SCM strain sensors. (**a**) Schematic illustration showing the different attachment modes of the SCM sensors to a PET substrate using the planar backside (**i**) and the frontside (**ii**) of the SCM. (**b**) Photograph showing the different adhesion behaviors of the SCM sensors attached to a flexible PET substrate using its backside and frontside under bending stress.

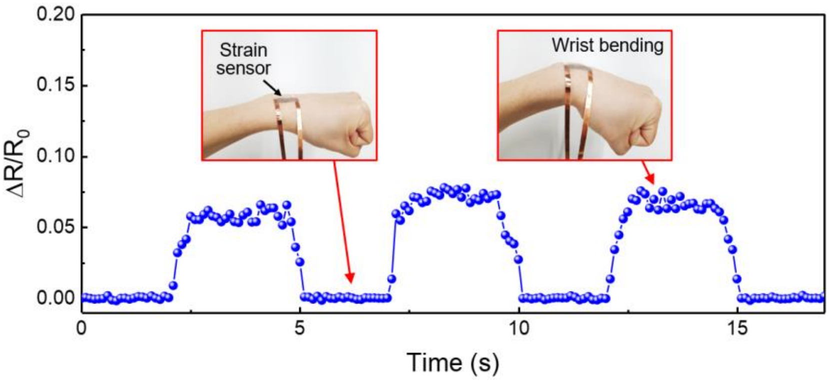

Figure S7. Demonstration of a self-attachable strain sensor for the monitoring application of human physical activities.

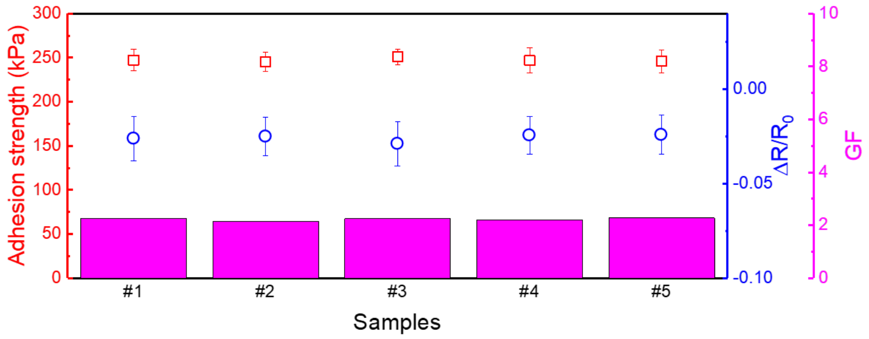

Figure S8. Adhesion strengths, relative resistance changes for a normal pressure (100 kPa), and GFs for tensile strains (range: 0 to 80%) of five SCM samples (coating dose of MWCNTs: 155.3 μg cm^{-2}). The average values and the error bars are based on 10 measurements. The error bars represent the standard deviations.

References

1. Oh, J.; Yang, J.C.; Kim, J.O.; Park, H.; Kwon, S.Y.; Lee, S.; Sim, J.Y.; Oh, H.W.; Kim, J.; Park, S. Pressure insensitive strain sensor with facile solution-based process for tactile sensing applications. *ACS Nano* **2018**, *12*, 7546–7553.

2. Kim, S.-R.; Kim, J.-H.; Park, J.-W. Wearable and transparent capacitive strain sensor with high sensitivity based on patterned Ag nanowire networks. *ACS Appl. Mater. Interfaces* **2017**, *9*, 26407–26416.
3. Drotlef, D.M.; Amjadi, M.; Yunusa, M.; Sitti, M. Bioinspired composite microfibers for skin adhesion and signal amplification of wearable sensors. *Adv. Mater.* **2017**, *29*, 1701353.
4. Fan, L.; Xie, J.; Zheng, Y.; Wei, D.; Yao, D.; Zhang, J.; Zhang, T. Antibacterial, self-Adhesive, recyclable, and tough conductive composite hydrogels for ultrasensitive strain sensing. *ACS Appl. Mater. Interfaces* **2020**, *12*, 22225–22236.
